# Endometrial carcinoma complicated by malignant pericardial effusion: A case report on the therapeutic regimen

**DOI:** 10.1097/MD.0000000000017584

**Published:** 2019-10-18

**Authors:** Guang Liu, Qianqian Zhang, Ze Li, Xiaojun Chen, Ning Zhang, Jinli Zhang

**Affiliations:** a Department of Cardiology, Hebei Medical University Fourth Affiliated Hospital and Hebei Provincial Tumor Hospital; b Graduate School of Hebei Medical University; c Department of Emergency, Hebei Medical University Second Affiliated Hospital, Shijiazhuang, Hebei, P. R. China.

**Keywords:** endometrial carcinoma, pericardial metastasis, treatment

## Abstract

**Rationale:**

High-stage endometrial carcinoma is an aggressive tumor with a high propensity for distant spread. However, metastases to the pericardium are rare in gynecological cancer, and are usually fatal.

**Patient concerns:**

A 69-year-old woman was diagnosed with endometrial carcinoma with pericardium metastasis. The symptoms at presentation were panic and shortness of breath.

**Diagnoses:**

The cytologic examination of pericardial fluid obtained by pericardiocentesis confirmed metastasis.

**Interventions:**

In addition to cisplatin instilled into the pericardial space, for systemic chemotherapy, we chose that gemcitabine and lobaplatin regimen be preferred.

**Outcomes:**

The patient has been participating in telephone follow-up for 8 months and has generally remained in a good condition.

**Lessons:**

Endometrial carcinoma can have pericardial metastases. When this happens, we recommend ultrasound-guided pericardial puncture and the pericardial injection of cisplatin, in combination with systemic chemotherapy that consists of gemcitabine and lobaplatin.

## 1 Introduction

Endometrial carcinoma is the most common gynecological cancer in developed countries. More than 90% of cases of endometrial cancer occur in women >50 years of age, with a median age at diagnosis of 63 years.^[1]^ Multiple risk factors for endometrial cancer have been identified: the early onset of menstruation, obesity, nulliparity, late menopause, diabetes mellitus, hypertension, infertility, unopposed estrogen exposure tamoxifen, and genetic syndromes.^[2]^ Endometrial carcinoma metastasis is common, particularly at high surgical stages and grades, and the more frequent metastatic sites include lymph nodes, omentum, lungs, and liver.^[3]^ However, pericardium metastasis for endometrial cancer is very rare. Here we report a case of endometrial adenocarcinoma cardiac metastasis that presented as malignant pericardial effusion with cardiac tamponade. The patient has remained in good condition throughout the treatment.

## 2 Case report

**Figure 1:**
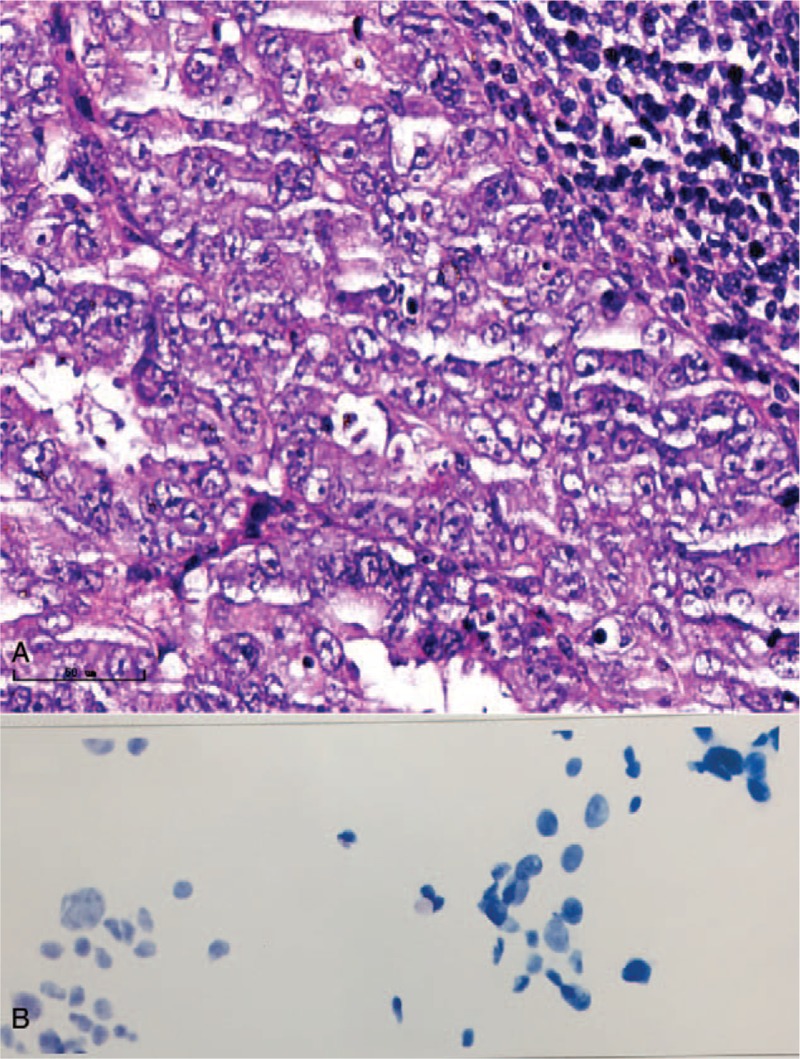
(A) Histologic examination of the endometrial specimen reveals a poorly differentiated endometrial adenocarcinoma. (B) The cytologic examination of pericardial fluid identifies tumor cells.

**Figure 2:**
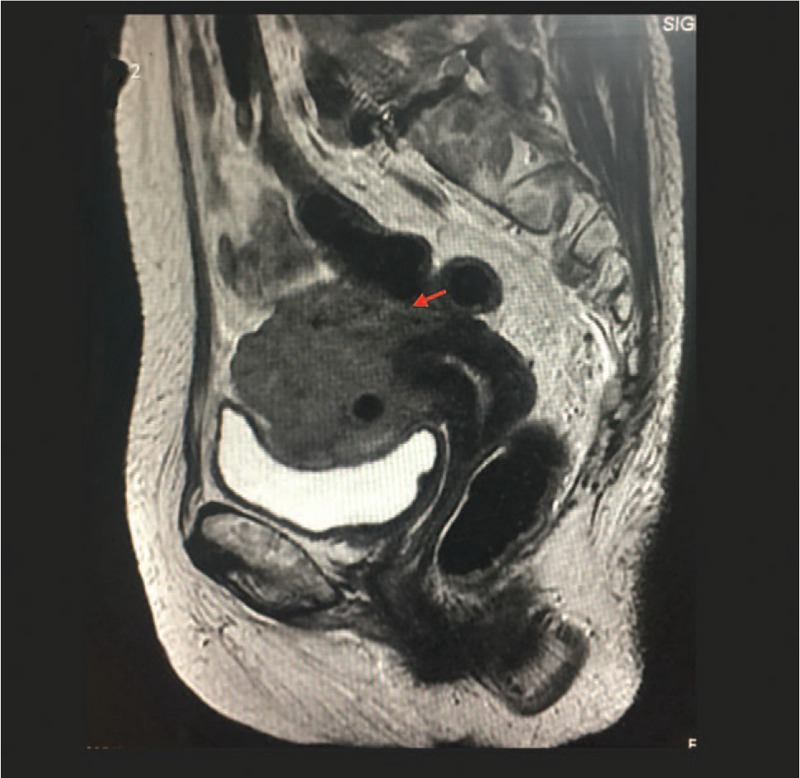
MRI reveals direct invasion of the rectal mucosa.

**Figure 3:**
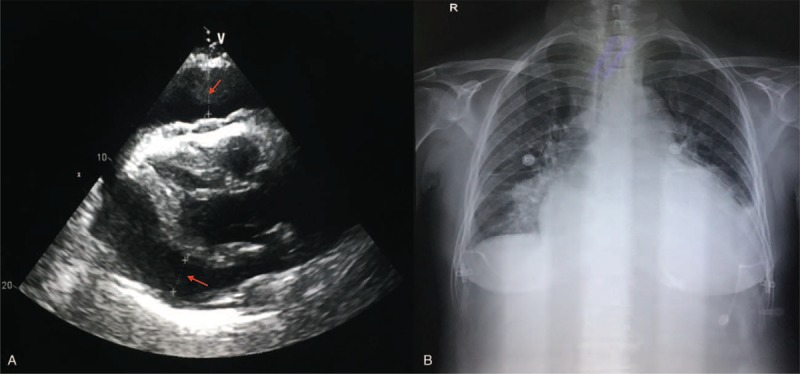
(A) Echocardiogram shows a large amount of pericardial effusion. (B) The chest X-ray reveals prominent cardiomegaly, with evidence of pericardial effusion.

A 69-year-old woman, gravida 4, para 3, was admitted to the hospital for curettage because of vaginal bleeding in January 2017. The histologic examination of the endometrial specimen revealed a poorly differentiated (grade 3) endometrioid-type endometrial adenocarcinoma (Fig. 1A). Immunohistochemical staining showed diffuse positivity of the neoplastic cells for cytokeratin, vimentin, carcinoembryonic antigen, p16, estrogen receptor alpha, progesterone receptor, and p53. Magnetic resonance imaging (MRI) revealed direct invasion of the rectal mucosa, and she was staged as IVA (Fig. 2). Systemic chemotherapy (paclitaxel and carboplatin) was given for 4 courses, with 28-day intervals, until May 2017. On June 25, 2017, she was admitted to our hospital due to panic and shortness of breath, which became heavier after activity, adopting the supine position. The physical examination revealed bilateral jugular vein dilatation and increased heart rate, on both sides. The echocardiogram confirmed the presence of a large amount of pericardial fluid (Fig. 3A), and the chest X-ray revealed a prominent cardiomegaly, with evidence of pericardial effusion (Fig. 3B). Thoracocentesis was performed, a pericardial catheter was placed, and 900 mL of fluid was drained. The cytologic examination of the pericardial fluid identified the presence of tumor cells (Fig. 1B). The patient was diagnosed with endometrial cancer after chemotherapy, with pericardial metastases.

**Figure 4:**
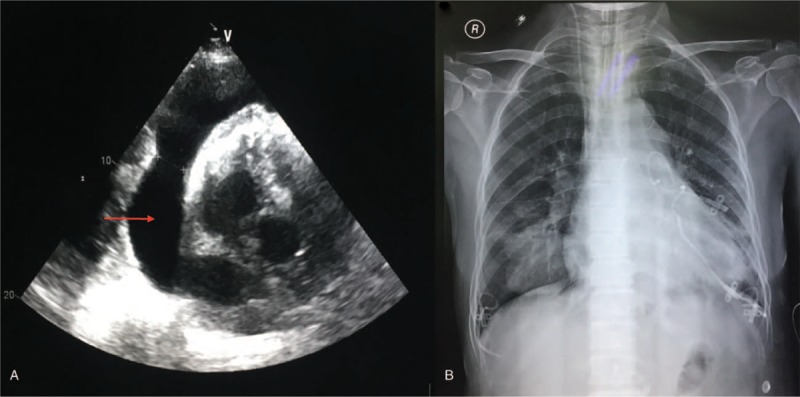
(A) Echocardiogram confirms the presence of a large pericardial effusion. (B) The chest X-ray reveals an increase in heart shadow.

Then, cisplatin was instilled into the pericardial space on July 1 (20 mg), July 4 (40 mg), and July 9 (20 mg), and systemic chemotherapy that consisted of cyclophosphamide and carboplatin was given over 2 courses, until September 20, 2017. However, 6 weeks later, she was admitted to our hospital for the same symptoms that were previously observed. The echocardiogram confirmed the presence of a large pericardial effusion (Fig. 4A), and the chest X-ray revealed an increase in heart shadow (Fig. 4B). We performed a thoracentesis, placed the catheter, and drained 700 mL of fluid. These findings indicated that the last treatment did not control the pericardial effusion (according to RECIST v1.1 criteria).^[4]^ Therefore, we again instilled cisplatin with a dose of 20, 40, and 20 mg into the pericardial space on November 4, 7, and 11, respectively. Furthermore, systemic chemotherapy with gemcitabine (1.2 g, d1 and d8) and lobaplatin (30 mg, d1) was given over 4 courses, with 28-day intervals, until March 10, 2018. No obvious adverse reactions occurred during the chemotherapy, and the patient was continuously followed up by telephone for 8 months and had generally remained in a good health condition (http://ctep.cancer.gov/forms/CTCAEv3.pdf).

This study complied with the Declaration of Helsinki and was approved by the Human Ethics and Research Ethics Committees of the Fourth Hospital of Hebei Medical University. Informed written consent was obtained from the patient for publication of this case report and accompanying images.

## 3 Discussion

Malignancy is the most common cause of pericardial effusion.^[5]^ Hematopoietic malignancies are the most frequent cause of malignant effusion, followed by lung cancer, breast cancer, and malignant melanoma.^[6]^ Under the physiological state, there is 25 to 50 mL liquid in the pericardium, which can be detected by ultrasound when >50 mL. However, normal compliance pericardium may be able to slowly collect up to 2 L of fluid without obvious symptoms before signs of tamponade appear.^[7]^ When symptoms occur, they include shortness of breath, dyspnea (exertional), orthopnea, chest discomfort or chest pain, cough, malaise, weakness, fatigue, peripheral edema, palpitations, hemoptysis, nausea, and vomiting. The most common body findings are pulsus paradoxus, tachycardia, peripheral edema, elevated jugular pressure, hypotension, pericardial rub, and decreased heart sounds.^[8]^ When patients develop these symptoms or signs, doctor should consider pericardial effusion. Confirmation of the diagnosis is primarily made through echocardiography, which allows full assessment of the hemodynamic effects of the effusion and also allows serial monitoring of the effusion before/after treatment.^[9]^ When the volume of malignant pericardial effusion exceeds 60 mL,^[6]^ pericardial puncture and exfoliation should be performed in time to detect the exfoliation of the drainage and determine whether cancer cells are present.

Mukai K et al evaluated 2177 patients with endometrial carcinoma between 2006 and 2016 and found that locoregional relapse included recurrences in the regional pelvic lymph nodes (1.0%) and the vaginal vault (4.5%), whereas distant recurrences included lung (including pleura and lymphangitic carcinomatosis) (3.4%), peritoneal (1.6%), liver (1.4%), distant lymph node metastases (beyond the regional lymph nodes) (1.2%), bone (including bone marrow) (1.1%), and brain (0.8%).^[10]^ This study did not report pericardial metastasis. We reviewed previous literature and found that only 5 cases of endometrial carcinoma with pericardial metastasis have been reported (Table 1). Overall, the prognosis of these patients was very poor after diagnosis of pericardium metastasis (survival of 17–173 days). However, our patient has lived >240 days after proper treatment. Reports demonstrated that gemcitabine may reverse the resistance mechanism of cisplatin in endometrial cancer by regulating the expression of glutathione (GSH)/glutathione-S-transferase (GST) and phosphorylation of p53, increasing cytotoxic activity.^[16,17]^ In addition, Tanaka et al treated 30 patients with advanced or recurrent endometrial cancer with gemcitabine, levofloxacin, irinotecan, and 5-fluorouracil (5-FU) (GLIF). The response and disease control rate were 7.1% and 39.3%, respectively, and the median survival was 3 months.^[18]^ Moreover, some similar studies showed that gemcitabine and cisplatin in patients with advanced endometrial cancer had a disease response rate of 50%.^[19,20]^ Furthermore, it is notable that most of the single-center small-scale studies of clinical trials on gemcitabine lack scientific rigor, the data seem to be less stringent, and sometimes controversial conclusions can be found.^[21]^

**Table 1:**
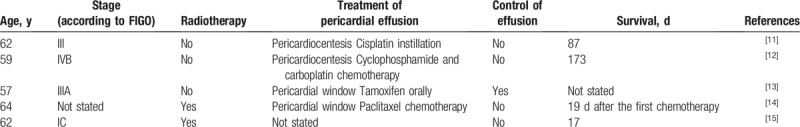
Review of the previously reported cases of endometrial carcinoma with pericardial metastasis.

Currently, there is no very effective treatment for patients with advanced endometrial carcinoma. Prognosis remains poor for those patients, despite receiving chemotherapy.^[22]^ However, if cardiac tamponade symptoms occur, ultrasound-guided pericardial puncture is currently the simplest and most effective method.^[23]^ On the basis of our experience in treating this patient, we recommend the pericardial injection of cisplatin, in combination with systemic chemotherapy that consists of gemcitabine and lobaplatin (second-line chemotherapy^[18]^) instead of cyclophosphamide and carboplatin (first-line chemotherapy).

## Author contributions

**Conceptualization:** Ning Zhang, Jinli Zhang.

**Data curation:** Xiaojun Chen.

**Formal analysis:** Ze Li.

**Software:** Ze Li, Ning Zhang.

**Writing – original draft:** Guang Liu.

**Writing – review and editing:** Qianqian Zhang, Jinli Zhang.

## References

[1] Colombo N Creutzberg C Amant F ESMO-ESGO-ESTRO consensus conference on endometrial cancer: diagnosis, treatment and follow-up. Radiother Oncol 2015;117:559 81. 26683800 10.1016/j.radonc.2015.11.013

[2] Colombo N Preti E Landoni F Endometrial cancer: ESMO Clinical Practice Guidelines for diagnosis, treatment and follow-up. Ann Oncol 2011;22 suppl. 6:vi35 9. 21908501 10.1093/annonc/mdr374

[3] Makris GM Mene J Battista MJ Endometrial carcinoma with tibial bone metastasis: a case report and literature review. J Obstet Gynaecol 2018;1 9. 10.1080/01443615.2017.1420759 29884070

[4] Eisenhauer EA Therasse P Bogaerts J New response evaluation criteria in solid tumours: revised RECIST guideline (version 1.1). Eur J Cancer 2009;45:228 47. 19097774 10.1016/j.ejca.2008.10.026

[5] Gornik HL Gerhard-Herman M Beckman JA Abnormal cytology predicts poor prognosis in cancer patients with pericardial effusion. J Clin Oncol 2005;23:5211 6. 16051963 10.1200/JCO.2005.00.745

[6] Kalogeraki A Lazopoulos G Papadakis GZ Cytology of pericardial effusion due to malignancy. Rom J Intern Med 2016;54:179 83. 27658166 10.1515/rjim-2016-0026

[7] Rieke JW Kapp DS Successful management of malignant pericardial effusion in metastatic squamous cell carcinoma of the uterine cervix. Gynecol Oncol 1988;31:338 51. 3049263 10.1016/s0090-8258(88)80013-1

[8] Burazor I Imazio M Markel G Malignant pericardial effusion. Cardiology 2013;124:224 32. 23571453 10.1159/000348559

[9] Ghosh AK Crake T Manisty C Pericardial disease in cancer patients. Curr Treat Options Cardiovasc Med 2018;20:60. 29936603 10.1007/s11936-018-0654-7 PMC6015600

[10] Mukai K Shinkai T Tominaga K The incidence of secondary tumors of the heart and pericardium: a 10-year study. Jpn J Clin Oncol 1988;18:195 201. 3411785

[11] Hayashi Y Iwasaka T Hachisuga T Malignant pericardial effusion in endometrial adenocarcinoma. Gynecol Oncol 1988;29:234 9. 3338674 10.1016/0090-8258(88)90218-1

[12] Santala M Puistola U Kauppila A Endometrial adenocarcinoma complicated by malignant pericardial effusion. Gynecol Oncol 1995;56:444 5. 7705682 10.1006/gyno.1995.1078

[13] Kheterpal P Singh M Mondul A Malignant pericardial effusion and cardiac tamponade in endometrial adenocarcinoma. Gynecol Oncol 2001;83:143 5. 11585427 10.1006/gyno.2001.6340

[14] Ramirez PT Ramondetta LM Burke TW Metastatic uterine papillary serous carcinoma to the pericardium. Gynecol Oncol 2001;83:135 7. 11585425 10.1006/gyno.2001.6351

[15] Castillo-Sang M Slam K Gociman B Endometrial adenocarcinoma metastatic to the right ventricle: a case report and review of the literature. Cardiovasc Pathol 2009;18:178 82. 18402828 10.1016/j.carpath.2007.12.007

[16] Smith JA Brown J Martin MC An in vitro study of the inhibitory activity of gemcitabine and platinum agents in human endometrial carcinoma cell lines. Gynecol Oncol 2004;92:314 9. 14751176 10.1016/j.ygyno.2003.09.037

[17] Smith JA Gaikwad A Ramondetta LM Determination of the mechanism of gemcitabine modulation of cisplatin drug resistance in panel of human endometrial cancer cell lines. Gynecol Oncol 2006;103:518 22. 16690105 10.1016/j.ygyno.2006.03.042

[18] Tanaka Y Ueda Y Nakagawa S A phase I/II study of GLIF combination chemotherapy for taxane/platinum-refractory/resistant endometrial cancer (GOGO-EM2). Cancer Chemother Pharmacol 2018;82:585 92. 30030584 10.1007/s00280-018-3648-y PMC6132850

[19] Brown J Smith JA Ramondetta LM Combination of gemcitabine and cisplatin is highly active in women with endometrial carcinoma: results of a prospective phase 2 trial. Cancer 2010;116:4973 9. 20665499 10.1002/cncr.25498 PMC4210375

[20] Eichbaum MH Sohn C Combination of gemcitabine and cisplatin is highly active in women with endometrial carcinoma: results of a prospective phase 2 trial. Cancer 2011;117:3529 author reply 3529-3530. 21287533 10.1002/cncr.25923

[21] Tait DL Blessing JA Hoffman JS A phase II study of gemcitabine (gemzar, LY188011) in the treatment of recurrent or persistent endometrial carcinoma: a gynecologic oncology group study. Gynecol Oncol 2011;121:118 21. 21159366 10.1016/j.ygyno.2010.11.027

[22] Smogeli E Cvancarova M Wang Y Clinical Outcome of Patients With High-Risk Endometrial Carcinoma After Treatment With Chemotherapy Only. Int J Gynecol Cancer 2018;28:1789 95. 30365455 10.1097/IGC.0000000000001356

[23] Virk SA Chandrakumar D Villanueva C Systematic review of percutaneous interventions for malignant pericardial effusion. Heart 2015;101:1619 26. 26180077 10.1136/heartjnl-2015-307907

